# Bodily experiences in multiple sclerosis: functional neurological symptoms, body image dissatisfaction, and disordered eating behaviors. A narrative review

**DOI:** 10.3389/fpsyg.2026.1890987

**Published:** 2026-09-09

**Authors:** Samuela Tarantino, Martina Proietti Checchi, Laura Papetti, Gabriele Monte, Michela Ada Noris Ferilli, Massimiliano Valeriani

**Affiliations:** 1 Developmental Neurology Unit, Bambino Gesù Children's Hospital, Istituto di Ricovero e Cura a Carattere Scientifico (IRCCS), Rome, Italy; 2 Systems Medicine Department, Tor Vergata University of Rome, Rome, Italy; 3 Translational Pain Neuroscience and Precision Medicine, CNAP, Department of Health Science and Technology, School of Medicine, Aalborg University, Aalborg, Denmark

**Keywords:** bodily experience, body-image disturbance, disordered eating, functional neurological symptoms, multiple sclerosis

## Abstract

Multiple sclerosis (MS) is a chronic disease of the central nervous system, associated with a range of physical, cognitive, and psychological difficulties. Beyond classical neurological symptoms, disturbances in bodily experience, including functional neurological symptoms, somatization, body-image disturbances, and disordered eating behaviors, represent an underrecognized, yet clinically relevant dimension of the disease. This narrative review synthesizes evidence from adult and pediatric populations, highlighting the frequent co-occurrence of these disturbances with MS and their associations with psychological symptoms, reduced quality of life, and functional impairment. Across studies, these manifestations appear to be shaped by a complex interplay of psychological vulnerability (e.g., depression, anxiety, perceived stigma, and low self-esteem), disease-related characteristics (such as disability level and disease course), and contextual factors including stress and treatment experiences, rather than being solely attributable to neurological pathology, such as demyelinating lesions, axonal damage, or brain atrophy. Notably, the relationship with objective disease markers is often modest or inconsistent, underscoring the importance of considering non-organic contributors to bodily experience. The limited available literature suggests that disturbances in bodily experience represent a clinically relevant yet underexplored dimension of MS, particularly in pediatric populations, warranting greater attention in both research and clinical assessment.

## 1 Introduction

Multiple sclerosis (MS) is a chronic autoimmune and neuroinflammatory disease of the central nervous system characterized by demyelination, neurodegeneration, and a heterogeneous spectrum of physical and cognitive impairments, including motor dysfunction, sensory disturbances, fatigue, and cognitive decline (Jellinger, 2024; Mey et al., 2023; Mohammed, 2024). Although MS typically presents in adulthood, pediatric-onset MS (POMS) accounts for approximately 3%−5% of cases and carries unique developmental implications, including cognitive, behavioral, and academic functioning (Brola and Steinborn, 2020; Fisher et al., 2020; Tarantino et al., 2024). Beyond neurological symptoms, MS strongly influences psychological functioning, emotional wellbeing, and overall quality of life (Gil-González et al., 2020; Göksel Karatepe et al., 2011; McKay et al., 2020). MS also affects multiple dimensions of bodily experience, encompassing the perception and interpretation of bodily sensations, the subjective experience and evaluation of the body, and body-related behaviors. Among these, functional neurological symptoms (FNS) and somatization, body image disturbance (BID), and disordered eating behaviors (DEBs) represent clinically relevant manifestations of altered bodily experience, with potential implications for psychological wellbeing and daily functioning (American Psychiatric Association, 2022; Prnjak et al., 2022; Conceição and Goldschmidt, 2019). Psychological comorbidities are particularly frequent in MS, with mood and anxiety disorders affecting approximately 30% of individuals with MS (Marrie et al., 2015; Peres et al., 2022; Zhang et al., 2023). Based on available evidence, similar psychological difficulties are also reported in POMS populations, indicating that these challenges extend across developmental stages (Fisher et al., 2020; Tarantino et al., 2024). These symptoms often interact with physical limitations such as fatigue, pain, and reduced mobility, producing reciprocal effects that exacerbate distress and complicate disease management (Brown et al., 2008; Marck et al., 2017). Across chronic medical conditions affecting both pediatric and adult populations, persistent physical symptoms and alteration in bodily functioning can heighten attention to internal bodily sensations and increase somatic focus (Cerutti et al., 2017; Locatelli et al., 2023; Wolters et al., 2022). Alterations in bodily awareness or a diminished sense of bodily control may further intensify this focus, increasing the likelihood of misinterpreting bodily sensations (Locatelli et al., 2023; Wolters et al., 2022).

From a neurocognitive perspective, interoceptive processing is supported by distributed brain networks involved in the integration and interpretation of bodily signals, including the insula, anterior cingulate cortex, thalamus, and parietal regions. These systems are involved in the continuous updating of internal bodily states, and emerging evidence suggests that alterations within these networks may be associated with changes in bodily awareness and symptom salience (Salamone et al., 2018; Gonzalez Campo et al., 2020).

In MS lesion burden and inflammatory activity may be associated with alterations in these interoceptive and sensorimotor networks, potentially contributing to changes in bodily signal processing (Carotenuto et al., 2020, 2023; Raimo et al., 2024; Gonzalez Campo et al., 2020). Such alterations may, in turn, be linked to increased attentional focus on bodily sensations and to the misinterpretation of ambiguous or benign physical signals. At a further level, MS-related neuroinflammatory processes may also influence broader neuromodulatory systems, including dopaminergic and serotonergic signaling. These systems are involved in the regulation of interoceptive, affective, and motivational processes, and their dysregulation has been proposed to contribute to fatigue, emotional disturbances, and altered reward processing in MS (Courtiol et al., 2021; Carotenuto et al., 2023).

One important dimension of bodily experience concerns the perception and interpretation of physical symptoms. FNS and somatization refer to alterations in symptom perception and expression, characterized by heightened attention to bodily sensations and an increased tendency to experience or report symptoms that may not be fully explained by underlying disease mechanisms alone (American Psychiatric Association, 2022; Espay et al., 2018; Stone et al., 2005; Kroenke, 2003; Lipowski, 1988). In MS, symptoms such as fatigue, sensory disturbances, pain, and visual or motor difficulties may amplify somatic vigilance and, in some cases, contribute to somatization (i.e., the tendency to express psychological distress through physical complaints) or functional neurological symptoms (i.e., neurological symptoms inconsistent with recognized disease mechanisms), particularly when reported symptom severity is disproportionate to objective clinical or neurophysiological findings (American Psychiatric Association, 2022; Kroenke, 2003; Espay et al., 2018; Stone et al., 2005; Walzl et al., 2022; Piliavska et al., 2023). Although these conditions are diagnostically distinct, they are often considered within a shared framework of symptom perception due to overlapping cognitive and perceptual features, including heightened attention to bodily sensations and increased likelihood of interpreting ambiguous interoceptive signals as signs of dysfunction (Stone et al., 2005; Wolters et al., 2022). This shared perspective is broadly consistent with interoceptive and predictive processing accounts of symptom perception, which suggest that prior expectations may influence the experience and interpretation of bodily sensations (Wolters et al., 2022).

Another important dimension of bodily experience concerns the subjective experience and evaluation of the body. BID refers to negative perceptions, thoughts, and feelings about one's own body, which may influence self-esteem, psychosocial adjustment, and overall wellbeing (Prnjak et al., 2022).

Changes in physical functioning, appearance, and activity limitations can negatively affect body satisfaction, self-esteem, and psychosocial adjustment (Barbagallo et al., 2024; Gholizadeh et al., 2019; Kumar, 2023; Liu et al., 2019; Pinquart, 2013; Senkowski and Heinz, 2016). These mechanisms are also relevant to MS, since the unpredictability of symptoms, alterations in physical condition, and treatment-related effects may shape body image and influence the subjective experience of the body (Lo Buono et al., 2023; Breheny et al., 2026; Pfaffenberger et al., 2011).

A related, yet distinct, dimension of bodily experience concerns eating-related attitudes and behaviors. DEBs encompass maladaptive eating attitudes or behaviors, such as restrictive eating, binge eating, or compensatory behaviors, that may occur without fulfilling the diagnostic criteria for an eating disorder (American Psychiatric Association, 2022; Conceição and Goldschmidt, 2019).

Treatments such as corticosteroids, which can induce weight gain or change body composition, may further contribute to body dissatisfaction and lower self-esteem, potentially increasing the risk of disordered eating behaviors (DEBs) (Fornari et al., 2001). These considerations are particularly salient in POMS, which emerges during a developmental period characterized by heightened self-awareness, social comparison, and sensitivity to perceived physical differences (MacAllister et al., 2007; Novak, 2025). Adolescents with MS must navigate disease-related bodily changes alongside puberty and identity formation, which may intensify emotional distress, somatic concerns, and body image difficulties (Novak, 2025).

Despite growing interest in bodily experience in MS, research on somatization, functional symptoms, body image, and eating behaviors remains limited, especially in pediatric populations. This gap highlights the importance of examining how MS affects multiple dimensions of bodily experience across the lifespan and their impact on psychological wellbeing and daily functioning.

### 1.1 Aims

This review aims to synthesize current evidence on MS by addressing: (1) FNS and somatization, as well BID and DEBs, highlighting how these aspects shape patients' bodily experience; and (2) potential factors associated with these difficulties, including disease-related, psychological, social, and life-span variables, to provide a comprehensive understanding of the mechanisms influencing patients' experiences across the lifespan.

## 2 Methods

### 2.1 Search strategy

This narrative review synthesizes current evidence on psychological manifestations involving bodily experience in MS, with a specific focus on FNS, somatization, BID, and DEBs across the lifespan. A narrative approach was adopted due to the considerable heterogeneity in study designs, assessment methods, sample characteristics, and outcome measures, as well as the predominance of small samples and case reports in this research area. The primary aim was to provide a comprehensive qualitative synthesis of the available evidence. The literature search and study selection followed predefined procedures while maintaining the interpretative nature of a narrative review. This review adopts a structured narrative approach with systematic search and screening procedures, balancing methodological transparency with interpretative flexibility.

The literature was comprehensively searched in PubMed and Web of Science. These databases were selected because they provide broad and complementary coverage of biomedical, neurological, and psychosocial research, including studies conducted in both adult and pediatric MS populations. Considering the substantial overlap in indexing with other major databases, these two sources were considered sufficient to capture the relevant peer-reviewed literature for the purposes of this narrative review. Only peer-reviewed publications written in English were included. Studies published up to March 2026 were analyzed.

Search terms combined keywords related to MS and the specific symptom-related and body-related outcomes examined in this review, including:

- Population: “multiple sclerosis,” “pediatric-onset MS”
- Functional symptoms: “functional neurological symptoms,” “functional neurological disorder,” “somatization”
- Body-image related outcomes: “body image disturbances,” “body dissatisfaction”
- Eating-related outcomes: “disordered eating,” “eating disorders”

An example search string used was:

(“multiple sclerosis” OR “pediatric-onset MS”) AND (“functional neurological symptoms” OR “functional neurological disorder” OR “somatization” OR “body image disturbances” OR “body dissatisfaction” OR “disordered eating” OR “eating disorders”).

In addition, manual screening of the reference lists of relevant reviews and empirical studies was conducted to identify additional potentially eligible publications.

### 2.2 Inclusion and exclusion criteria

Studies were considered eligible if they included adults or children/adolescents diagnosed with MS, regardless of clinical subtype, and reported on functional neurological symptoms, somatization, BID, or BEDs. Eligible studies comprised empirical research and peer-reviewed case reports describing clinically relevant manifestations. Case reports were included when peer-reviewed and indexed, given their potential relevance in capturing rare or complex clinical presentations in MS populations. Studies were excluded if they focused exclusively on neurological, pharmacological, or rehabilitation outcomes without addressing symptom-related aspects connected to bodily experience, or if they examined eating behaviors solely in relation to metabolic, nutritional, or dietary interventions. Conference abstracts, editorials, commentaries, and non-peer-reviewed publications were also excluded. Studies including small samples ( ≤ 30 participants) were considered with caution; however, they were retained when they provided clinically meaningful insights into the phenomena of interest in MS.

### 2.3 Study selection

The study selection process was conducted in two phases.

#### 2.3.1 Phase 1—Title and abstract screening:

Duplicate records identified through database searches were first removed to ensure that only unique articles were considered. Two independent reviewers (S.T. and M.P.C.) screened the titles and abstracts of the remaining records to identify studies potentially relevant to FNS, somatization or body image-related constructs in MS populations. Screening explicitly included relevance to BID as part of the eligibility criteria. Articles clearly unrelated to these domains were excluded at this stage. When the relevance of a study was unclear, or when disagreements arose between the two reviewers, the article was retained for full-text evaluation.

#### 2.3.2 Phase 2—Full-text screening:

**Figure 1:**
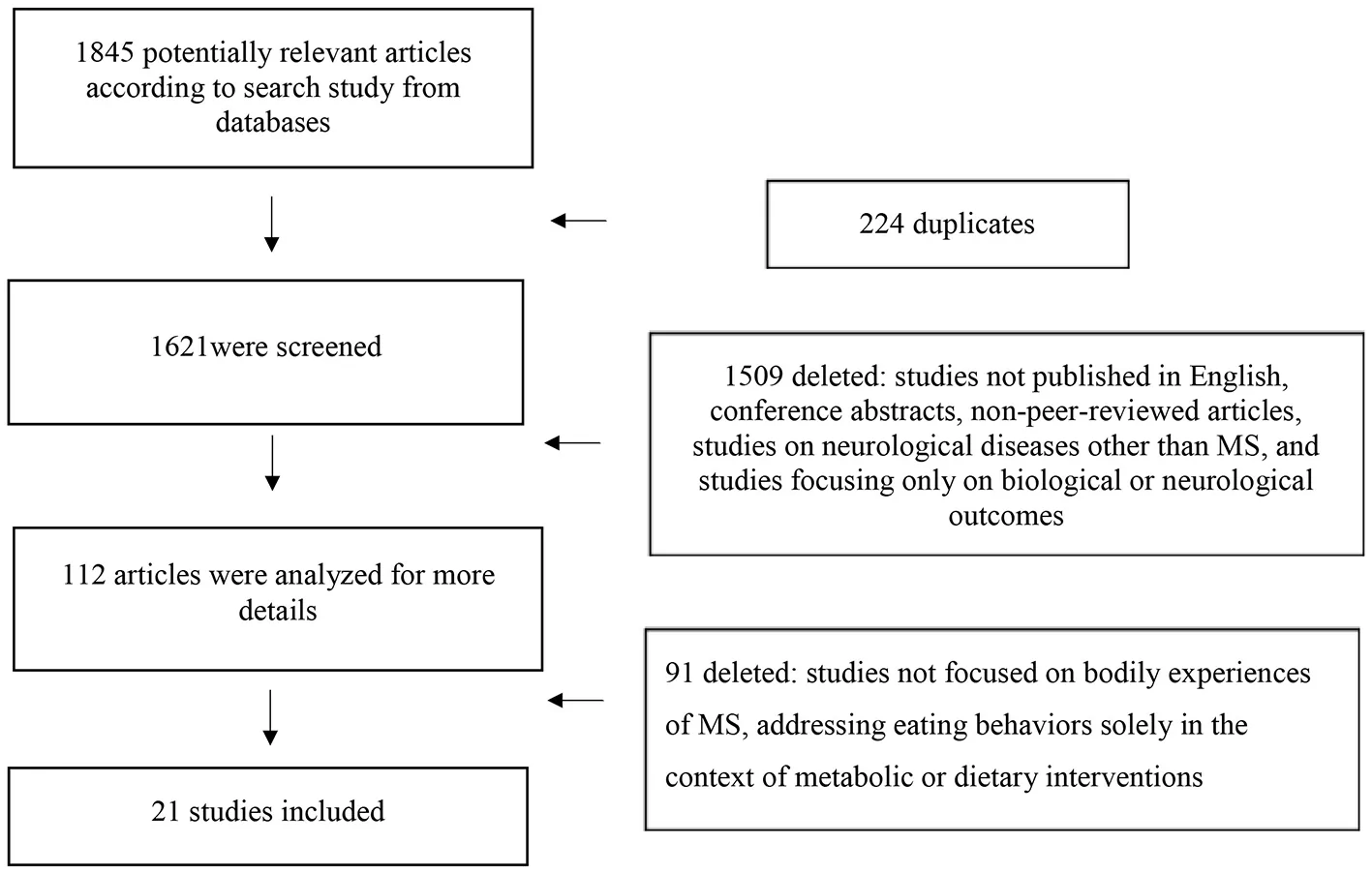
Flow diagram of the study methodology. Of the 21 reports considered, 19 were directly included studies and 2 were additional cases identified through cited references. Eighteen reports involved adults with MS and 3 pediatric/adolescent cases (< 18 years). By domain: FNS/somatization (*n* = 10), BID (*n* = 7), and DEBs (*n* = 4).

Three additional reviewers (L.P., G.M., and M.A.N.F.) independently assessed the full texts of the selected articles to confirm eligibility according to the predefined inclusion criteria. Studies that did not meet these criteria were excluded (Figure 1). Discrepancies among reviewers were resolved through discussion and consensus under the supervision of the senior author (M.V.). Given the narrative design of the review, no formal statistical measure of inter-rater reliability (e.g., Cohen's kappa) was calculated. Consequently, coding agreement was not quantified in terms of percentages; instead, agreement was achieved iteratively through discussion and consensus during the screening and coding process.

### 2.4 Data extraction and synthesis

For each included study, the following information was extracted:

- Sample characteristics (MS subtype, age, sample size, and presence of control groups).
- Outcomes assessed, including functional neurological symptoms, somatization, BID, and BEDs.
- Main findings related to psychological manifestations involving bodily experience.

The evidence was synthesized using a thematic narrative approach, distinguishing between adult MS populations and POMS.

The narrative synthesis followed three main steps:

1. Preliminary synthesis: studies were organized according to the principal thematic domains addressed in this review, including functional neurological symptoms and somatization, BID, and BEDs
2. Exploration of relationships: similarities and discrepancies across studies were examined, with particular attention to clinical and disease-related factors potentially associated with these phenomena.
3. Assessment of the synthesis: findings were qualitatively summarized, giving greater narrative weight to studies with larger samples or stronger methodological designs.

Case reports and studies with very small samples were also considered when they provided clinically relevant insights, although their limited generalizability was explicitly acknowledged.

No formal risk-of-bias or methodological quality assessment was conducted due to limited comparability across study types. Instead, the methodological characteristics of the included studies were considered during the narrative synthesis, based on extracted study characteristics (e.g., study design, sample size, population, and outcomes) reported in the summary table (Table 1). Given the narrative nature of this review, findings were integrated through a narrative synthesis approach.

**Table 1:** Summary of articles included in the review.

Author (year)	Sample	Study design	Study definition of constructs	Measures	Sample type and recruitment	Main findings	Limitations
Breheny et al. (2026)	104 MS patients diagnosis: RRMS 79.8%, PPMS 9.6%, SPMS 5.8% Age: 44.04 ± 11.33 years. 85 females (81.7%) 19 males (18.3%). 101 healthy controls Age: 38.43 ± 13.16 years. 84 females (83.2%) 17 males (16.8%).	Cross-sectional case-control study	Body image (appreciation and dissatisfaction); sexual functioning; interoception; psychological distress	MSISQ-15; BAS-2; BISS; MAIA-2; HADS; SF-36; PDDS	Community-based MS sample recruited through the MS Society of Ireland and social media	MS had more sexual dysfunction, lower body appreciation, higher anxiety/depression; body appreciation predicted sexual function	Cross-sectional design; self-report measures; predominantly female sample
Bruce and Arnett (2008)	53 MS patients diagnosis: RRMS 58%, SPMS 28%, PPMS 11%, PR 2% Age: 46.6 ± 7.6 to 49.5 ± 7.7 years 41 females (77%), 12 males (23%) Controls: none	Longitudinal observational study	Psychopathology; somatization; depression; anxiety; disability; cognitive functioning	SCL-90-R; BDI-II; CMDI; EDSS	Community-based MS sample recruited through neurologists and MS support groups)	Psychological distress largely stable; up to 52% had elevated symptoms; somatization and OCD most frequent; depression, anxiety, psychoticism also common; somatization correlated with disability	No control group; self-report measures; attrition over follow-up; not MS-specific
Carbonell et al. (2014)	Two pediatric MS patients. One patient aged 17 years; one with childhood-onset MS (first event at age 5 years, diagnosis at 13 years) Two females (100%) Controls: none	Case series	FNS (including functional relapses); psychological stress; anxiety; depression; MS-related disability	Clinical/neurological examination; brain and orbital MRI; visual evoked potentials; EDSS; psychiatric evaluation	Pediatric MS patients recruited from a tertiary MS center	Functional relapses mimicked MS relapses; inconsistent with exam/MRI; rapid or atypical recovery; associated with psychological stress; some patients also had true organic relapses	Very small sample; no control group; case series design; potential misclassification of functional and MS relapses
Charvet et al. (2016)	140 pediatric MS patients diagnosis: MS 77%, CIS 23% Age: 14.9 ± 2.6 years 81 females (57.9%) 59 males (42.1%) Controls: none	Cross-sectional observational study	Behavioral and emotional problems; somatization; fatigue; cognitive impairment; disability	BASC-2; EDSS; neuropsychological battery; Fatigue Severity Scale (subset)	Consecutive pediatric MS/CIS outpatients recruited from a specialized MS center	14.7% had significant somatization; common behavioral symptoms included attention problems and anxiety	Cross-sectional design; no control group; fatigue assessed in a subset; single-center sample
Cuoco et al. (2025)	310 MS patients diagnosis: All MS phenotypes ± FND Age: median 50 years (IQR 19) 224 females (72.3%) 86 males (27.7%) Controls: none	Prospective observational study	Comorbid FND in MS	Neurological examination (DSM-5); EDSS; AIS; clinician interview (anxiety/depression)	Consecutive MS patients recruited from a single MS center (elective and emergency visits)	5.8% had comorbid FND; FND mimicked relapses; higher depression/anxiety and disability overestimation in FND group	Cross-sectional design; no control group; predominantly female sample; no validated mood scales; possible underestimation of psychiatric symptoms
Jones et al. (2018)	513 MS patients diagnosis: Mixed types (55.9% RRMS) Age: 51.43 ± 10.98 years 419 females (81.7%) 94 males (18.3%) Controls: none	Cross-sectional observational study	Somatic symptom expression of depression and anxiety; influence of MS symptoms and disability on symptom measurement	PHQ-9; PRIME-MD Anxiety; self-report EDSS; MS symptom questionnaire	Community-dwelling people with MS recruited through the Greater Northwest Multiple Sclerosis Society and previous research studies	Somatic depressive symptoms common and largely independent of disease severity; fatigue, sleep, appetite changes frequent; anxiety symptoms partly influenced by MS; minimal misclassification of depression	Cross-sectional design; no control group; non-validated MS symptom questionnaire; no assessment of longitudinal changes, treatment effects, or early-stage disease
Kindrat (2007)	30 MS patients diagnosis: RRMS 30 females (100%) Controls: none	Cross-sectional descriptive correlational study	Body image; depression	BIQ; BDI-SF; Demographic questionnaire	Convenience sample recruited from an MS clinic	Body image and depression highly correlated; positive body image linked to less depression	Small sample; cross-sectional design; no control group; female-only sample
Kiropoulos et al. (2025)	275 MS patients diagnosis: RRMS 68.3%, Progressive 29.1% Age: 43.0 ± 12.9 years 218 females (79.3%) 55 males (20.0%) Two Non-binary (< 0.7%) Controls: none	Cross-sectional observational study	Body image dissatisfaction; DEBs; lifetime eating disorder history; neuroticism; self-esteem	BFI (neuroticism); RSES-10; BSQ-8; EAT-26; self-reported MS and SRDSS	Community-based MS sample recruited via online ads, support groups, and hospital noticeboards	21% lifetime eating disorder; neuroticism linked to low self-esteem and body dissatisfaction; mediated eating disorder symptoms	Cross-sectional design; no control group; self-report measures; convenience sample (selection bias); no clinical interview confirmation of eating disorder diagnosis
Lo Buono et al. (2023)	100 MS patients diagnosis: RRMS (all) Age: 40.3 ± 11.6 years 74 females (74.0%) 26 males (26.0%) Controls: none	Cross-sectional observational study	Body image dissatisfaction; self-esteem; psychopathology; physical disability	EDSS; BIS; RSES; SCL-90-R; Medication data extracted from clinical records	MS outpatients recruited from a neurology outpatient clinic	EDSS and age predicted body image concerns; psychopathology strongly correlated with body image and self-esteem	Cross-sectional design; no control group; RRMS only; self-report measures; single-center sample
Merwick and Sweeney (2008)	Three MS patients diagnosis: CDMS Age: 39, 39, 48 years Two females One male Controls: none	Case series	FNS; MS relapses	Neurological examination MRI CSF analysis Evoked potentials Clinical history	Patients recruited from emergency department and neurology outpatient services	Functional symptoms presented as MS pseudo-relapses, without clinical or radiological evidence of disease activity. Symptoms were often associated with psychological stressors and resolved rapidly	Very small sample; case series design; no control group; descriptive findings only
Pfaffenberger et al. (2011)	40 MS patients diagnosis: MS (RRMS, SPMS, PPMS not specified) Age: 37.35 ± 9.41 years 29 females (72.5%) 11 males (27.5%) 28 healthy controls Age: 35.82 ± 10.29 years 14 females (50.0 %) 14 males (50.0%)	Cross-sectional case-control study	Body image; depression; physical disability	FBeK, FKB, BDI, EDSS	Consecutive MS patients recruited from a neurology clinic; healthy controls recruited from hospital staff acquaintances	MS patients showed poorer body image than controls, including lower perceived attractiveness/self-confidence and greater concern about physical deficits; sexual problems higher in men	Cross-sectional design; no MS subtype detail
Piliavska et al. (2023)	234 MS patients diagnosis: RRMS 66.1%, PPMS 12.9%, SPMS 21% Age: 48.7 ± 8.95 years 168 females (71.8%) 66 males (28.2%) Controls: none	Cross-sectional observational study	FNS; health-related quality of life; work ability; neurological disability	Structured medical questionnaire; SF-36; EDSS	Consecutive MS inpatients recruited from a neurological rehabilitation hospital	17.1% showed symptoms not explained by MS structural pathology; FNS linked to worse quality of life and work ability	Cross-sectional design; no control group; single rehabilitation center; diagnosis of FNS partly based on clinical judgment; limited generalizability
Raimo et al. (2024)	59 MS patients diagnosis: RRMS 36, SPMS 23 Age: MS 46.88 ± 10.92 years 40 females (67.8%), 19 males (32.2%) 51 healthy controls Age: 46.04 ± 11.85 years 37 females (72.5%), 14 males (27.5%)	Cross-sectional case-control study	Body representations; interoception; disability	EDSS, BRB-N, ST, HL, FBE, HDT, SAQ, MoCA, HDRS, HARS	MS outpatients recruited from neurology outpatient clinics; healthy controls matched for age and education	MS patients had reduced interoceptive accuracy; RRMS increased visceral interoceptive sensibility; progressive MS slowed body schema response	Cross-sectional design; no neuroimaging; limited representation of MS subtypes
Samonds and Cammermeyer (1989)	20 MS patients diagnosis: MS with moderate-severe disability Age: 34–70 years (mean 47.61) 20 males (100%) Controls: none	Cross-sectional study	Body image satisfaction	BCS; BIS; EDSS	Clinical MS sample recruited from a hospital-based medical center	Body image satisfaction was unrelated to disability severity; older age and longer disease duration were associated with greater body/self-image satisfaction	Small sample; cross-sectional design; no control group; male-only veterans; outdated measures
Sarisoy et al. (2013)	76 MS patients diagnosis: RRMS 77.6%, Progressive 22.4% Age: MS 37.8 ± 10.2 years 56 females (73.7) 20 males (26.3%) 76 healthy matched controls Age: 37.8 ± 10.2 years	Cross-sectional case-control study	Eating attitudes; somatization; psychological distress; self-esteem	SCL-90-R, BDI, STAI-I/II, PSQI, PI, RSES, EAT, EDSS	Clinical MS sample recruited from a neurology outpatient clinic; healthy community controls	MS patients showed higher somatization and psychopathology than controls; progressive MS and higher disability were associated with worse psychiatric symptoms, lower self-esteem, and more disturbed eating attitudes	Cross-sectional design; no structured psychiatric interviews; single-center; self-report measures
Sengül et al. (2019)	50 MS patients diagnosis: RRMS Age: MS 31.66 ± 7.88 years 31 females (62%) 19 Males (38%) 45 Age- and sex-matched healthy controls	Cross-sectional study	Body image perception; anxiety; depression; clinical and demographic variables (MS patients without neurological disability)	BCS, BDI, BAI, EDSS	Consecutive MS patients recruited from a neurology outpatient clinic	Impaired body image perception despite minimal neurological disability; associated with marital status, disease onset, attacks, anxiety, depression	Cross-sectional design; self-report measures; no structured psychiatric interviews
Stevens et al. (2019)	151 MS patients diagnosis: RRMS 68.9%, SPMS 17.2%, PPMS 11.3%, CIS 2% Age: 46.1 ± 11.8 years 100 females (66.2%) 51 males (33.8%) Controls: none	Cross-sectional observational study	Body image dissatisfaction; depressive symptoms; anxiety; quality of life; clinical and demographic variables	BCS, PHQ-9, neuro-QoL, T25FW	MS patients recruited during outpatient visits or infusion appointments	Relatively low levels of body image dissatisfaction overall; women slightly higher BID; BID was associated with psychological and demographic variables, but not with MS clinical variables	Cross-sectional design; no control group; biased toward white female participants; self-report measures; limited subgroup power
Till et al. (2012)	31 pediatric MS patients diagnosis: pediatric RRMS Age: MS 16.1 years 23 females (74.2%) Eight males (25.8%) 31 healthy age matched controls	Cross-sectional observational study	Emotional and behavioral functioning; anxiety; depression; somatization; adaptive behaviors	BASC-2	Consecutive pediatric MS patients recruited from a pediatric MS clinic; community controls	24.1% of MS adolescents had clinically significant somatic symptoms; higher internalizing problems, depression, anxiety, functional difficulties vs controls; fatigue associated with worse somatic/behavioral symptoms	Cross-sectional design; reliance on self/parent report; mild disability
Timmerman and Stuifberg (1999)	67 MS patients diagnosis: RRMS 56.7%, Progressive MS 25.4%, Benign sensory MS 10.4% Age: 44.3 ± 10.5 years 67 females (100%) Controls: none	Cross-sectional observational study	Eating patterns and nutritional behaviors; depression; self-efficacy; social support; illness severity	3-day food diary; CES-D; PRQ-85; ISS	Community-dwelling MS sample recruited via community and support group channels	Altered eating patterns common; associated with fatigue, emotional distress, and coping with disease	Cross-sectional design; no control group; female-only sample; self-report bias

AIS, Athens insomnia scale; BAS2, body appreciation scale 2; BASC2, behavior assessment system for children 2; BAI, beck anxiety inventory; BCS, body cathexis scale; BDI, beck depression inventory; BDIII, beck depression inventory II; BDISF, beck depression inventory short form; BFI, big five inventory; BIQ, body image ideals questionnaire; BIS, body image scale; BRBN, brief repeatable battery of neuropsychological tests; BSQ8, body shape questionnaire 8; CDMS, clinically definite multiple sclerosis; CESD, Center for Epidemiologic Studies Depression Scale; CIS, clinically isolated syndrome; CMDI, Chicago multiscale depression inventory; CSF, cerebrospinal fluid; DSM5, Diagnostic and Statistical Manual of Mental Disorders, Fifth Edition; EAT, eating attitudes test; EAT26, eating attitudes test 26; EDSS, expanded disability status scale; FBE, fragebogen zur Körpererfahrung (body experience questionnaire); FBeK, fragebogen zur beurteilung des eigenen Körpers (questionnaire for assessment of one's own body); FKB, fragebogen zum körperbild (body image questionnaire); FND, functional neurological disorder; FNS, functional neurological symptoms; FSS, fatigue severity scale; HADS, hospital anxiety and depression scale; HARS, Hamilton anxiety rating scale; HDRS, Hamilton depression rating scale; HDT, hand laterality task; HL, heartbeat location/heartbeat detection task; IQR, interquartile range; ISS, illness severity scale; MAIA2, multidimensional assessment of interoceptive awareness 2; MoCA, montreal cognitive assessment; MRI, magnetic resonance imaging; MS, multiple sclerosis; MSISQ15, multiple sclerosis intimacy and sexuality questionnaire 15; NeuroQoL, quality of life in neurological disorders; OCD, obsessive compulsive disorder; PDDS, patient determined disease steps; PHQ9, patient health questionnaire 9; PI, personality inventory; PPMS, primary progressive multiple sclerosis; PRIMEMD, primary care evaluation of mental disorders; PRQ85, personal resource questionnaire 85; PSQI, Pittsburgh sleep quality index; RRMS, relapsing remitting multiple sclerosis; RSES, Rosenberg self esteem scale; RSES10, Rosenberg self esteem scale 10; SAQ, self awareness questionnaire; SCL90R, symptom checklist 90 revised; SF36, short form health survey 36; SPMS, secondary progressive multiple sclerosis; SRDSS, self reported disability status scale; ST, spatial task; STAI I/II, state trait anxiety inventory state and trait forms; T25FW, timed 25 foot walk.

## 3 Results

### 3.1 Functional neurological symptoms and somatization

Functional neurological symptoms (FNS) are neurological signs that are inconsistent with recognized disease mechanisms. When these symptoms meet formal DSM-5-TR criteria, they are classified as “Functional Neurological Disorder” (FND) (American Psychiatric Association, 2022; Espay et al., 2018; Stone et al., 2005). On the other hand, somatization, referred to in the DSM-5-TR as “Somatic symptom disorder,” reflects the tendency to express psychological distress through physical complaints (American Psychiatric Association, 2022; Kroenke, 2003; Lipowski, 1988). Medically unexplained symptoms are common in MS and can complicate diagnosis and management (Cuoco et al., 2025; Piliavska et al., 2023; Walzl et al., 2022). Case reports have long documented functional or non-organic neurological presentations in MS, emphasizing the difficulty of distinguishing these episodes from true inflammatory relapses. Early descriptions were first reported in the late 19th century, when Gowers (1893) reported “hysterical” symptoms in patients diagnosed with MS (cited in Walzl et al., 2022). More recently, functional symptoms, including, “pseudo-relapses,” have gained recognition as a clinically important phenomenon (Merwick and Sweeney, 2008; Ramo-Tello et al., 2021). Pseudo-relapses refer to transient exacerbations of pre-existing neurological deficits occurring in the absence of new inflammatory activity within the central nervous system and are commonly precipitated by systemic factors such as infection, psychological stress, or increased body temperature (Ramo-Tello et al., 2021).

In 2008, Merwick and Sweeney described three patients with clinically definite MS who presented with neurological symptoms mimicking relapse episodes. The cases included a 39-year-old man, and two women aged 39 and 48 years. These patients exhibited symptoms such as sudden weakness, sensory changes, and visual disturbances that were inconsistent with their underlying neurological disease and resolved rapidly without changes in objective neurological findings.

In the pediatric MS population, Carbonell et al. (2014) described functional relapses in two cases, characterized by neurological symptoms mimicking true MS relapses but not consistent with inflammatory demyelinating activity (Carbonell et al., 2014). In one case, a 17-year-old girl experienced recurrent episodes of weakness, numbness, and fatigue. While some episodes represented true MS relapses with corresponding clinical and MRI findings, others resolved rapidly without objective neurological or radiological evidence and followed self-reported psychological stress. The patient also reported significant anxiety about the long-term prognosis of MS and later received psychological counseling and pharmacological treatment for anxiety and depression. However, no formal psychiatric diagnosis or temporal association between psychological symptoms and neurological episodes was reported. The second case involved a girl diagnosed with POMS at age 13, presenting with recurrent optic neuritis. During one relapse, her right-eye symptoms were initially interpreted as a conversion disorder due to normal neurologic examination, MRI, and visual evoked potentials; later, findings confirmed right optic neuritis consistent with MS (Carbonell et al., 2014). Although objective ophthalmologic assessments demonstrated recovery, she continued to report visual loss, reflecting a functional overlay influenced by anxiety and depression.

Both received psychiatric support alongside disease-modifying therapy, highlighting the importance of recognizing functional symptoms in pediatric MS to ensure accurate diagnosis and appropriate management (Carbonell et al., 2014).

The understanding of FNS in MS has evolved over time, with research increasingly focused on quantifying their prevalence. In a sample of 234 MS patients, Piliavska et al. (2023) found that 55.1% of symptoms were completely lesion-based, while 17.1% were only partially or not at all attributable to structural lesions. Symptoms such as memory impairment, dysphagia, sexual dysfunction, speech disturbance, pain, and tremor could hardly be explained by lesions, whereas gait, balance, coordination, fine motor, and urinary symptoms were more consistently lesion-based. Emerging prospective data have clarified that FND in MS manifests in two primary forms: acute, relapse-like episodes (incident FND) and persistent, long-term functional symptoms. In a recent prospective cohort study by Cuoco et al. (2025) involving 310 adult MS patients, 5.8% were diagnosed with comorbid FND. Among patients admitted for suspected relapse, 22.6% (7/31) were identified as having incident FND, characterized by a sudden onset of functional symptoms mimicking an MS relapse. The remaining FND cases exhibited persistent functional symptoms, coexisting with established MS and contributing to ongoing functional disability. Depression, relapsing-remitting MS phenotype, and greater disability independently predicted the presence of FND. Acute FND was more frequent among younger patients and those with earlier disease onset, whereas persistent FND was associated with a long-term functional burden and disability.

These findings on FNS complement research on broader somatic manifestations in MS, highlighting that physical symptoms in this population often reflect both neurological and psychological contributions. In 2008, Bruce and Arnett observed stable psychopathology, including somatization, in 53 MS patients, with 26% showing clinical anxiety elevations and 52% elevated somatization. Subsequent studies have further highlighted elevated somatization in MS compared with healthy controls. Sarisoy et al. (2013) specifically reported higher somatization scores on the SCL-90-R questionnaire in MS patients (1.88 ± 0.91) vs. controls (0.94 ± 0.79), with a weak but positive correlation with the Expanded Disability Status Scale (EDSS). These observations are consistent with findings by Jones et al. (2018), who reported that somatic symptoms of depression in people with MS, such as fatigue, sleep disturbance, and appetite changes, are common but largely independent of disease severity, reflecting depressive processes rather than MS pathology.

Although data in pediatric MS are limited, available studies suggest similar patterns of somatization. Till et al. (2012) found elevated somatization scores in adolescents with POMS compared with healthy controls. Self-report measures indicated that 24.1% of MS participants endorsed clinically significant somatic symptoms, a pattern corroborated by parent ratings, which also highlighted increased somatic complaints and higher rates of depressive and anxiety symptoms. Similarly, Charvet et al. (2016) reported that among 140 children and adolescents with MS or clinically isolated syndrome, 29.9% of self-reports and 34.1% of parent reports indicated at least one clinically significant behavioral problem. Somatization was one of the most frequently elevated scales, with clinically significant somatic complaints reported by 14.7% of participants and 16.2% of parents.

### 3.2 Body-image disturbance

Body image is a multidimensional construct encompassing individuals' perceptions, attitudes, and emotions toward their body, including cognitive, affective, and behavioral components that extend beyond purely aesthetic concerns (Prnjak et al., 2022).

To date, no studies in MS have established the presence of BID according to formal DSM diagnostic criteria (American Psychiatric Association, 2022); rather, research has examined body image as a dimensional construct assessed through self-report measures. Although this line of research remains relatively limited and has primarily focused on adult populations, body dissatisfaction appears to be relatively common among individuals with MS.

Prevalence estimates of clinically significant BID range from 15% to 35%, depending on the assessment instruments and threshold criteria employed (Lo Buono et al., 2023; Stevens et al., 2019). Beyond prevalence, several studies have investigated the psychological and clinical factors associated with BID in MS. Within this framework, a number of studies have highlighted the strong association between BID and psychological distress (Lo Buono et al., 2023; Pfaffenberger et al., 2011; Stevens et al., 2019). (Pfaffenberger et al. 2011) reported robust links between body dissatisfaction and depressive symptoms, identifying BID as an important factor influencing psychological wellbeing and quality of life.

Early work suggested that negative body perceptions may increase psychological vulnerability independently of physical impairment (Kindrat, 2007). Evidence regarding the role of disease-related clinical variables remains inconsistent, as altered body image has been reported even among individuals with minimal or no objective neurological disability (Samonds and Cammermeyer, 1989; Sengül et al., 2019). In a study of 151 adults with MS, Stevens et al. (2019), found that higher body mass index (BMI), depressive symptoms, and perceived stigma were strongly associated with greater body dissatisfaction, particularly among women, whereas MS-specific variables- including treatment type, time since treatment, disease course, and functional performance- did not predict BID. These findings suggested that general psychosocial factors may play a more prominent role than disease-related characteristics. In contrast, other studies have reported a significant contribution of clinical variables. Lo Buono et al. (2023) extended these findings, exploring relationships between body image, self-esteem, psychological distress, and functional disability in a sample of 100 adults with relapsing-remitting MS. BID was strongly associated with psychological factors, as individuals with more negative body image reported higher levels of depression, anxiety, and global psychological distress, alongside markedly lower self-esteem. In addition to the prominent role of psychological factors, the study also highlighted the contribution of clinical and disease-related characteristics, with greater functional disability and higher EDSS scores associated with more severe BID.

The broader impact of BID extends beyond psychological wellbeing and may also affect functional and sexual health. In a study comparing 104 individuals with MS to 101 healthy controls, Breheny et al. (2026) found that body image and mood variables, together with disability status and illness duration, explained nearly half (48.6%) of the variance in sexual dysfunction. People with MS reported significantly greater sexual dysfunction, lower body appreciation, and higher body dissatisfaction than controls, highlighting the relevance of BID for sexual functioning in this population.

Despite these insights in adult populations, no studies to date have specifically investigated body-image disturbance in pediatric populations with MS. This gap underscores the need for further research to understand the psychosocial impact of MS on body perception during childhood and adolescence.

### 3.3 Disordered eating

DEBs encompass a range of irregular or dysfunctional eating patterns, including restrictive eating, binging, compensatory behaviors and irregular eating patterns. These behaviors may represent subclinical manifestations or may meet DSM-5-TR criteria for formal eating disorders (such as anorexia nervosa, bulimia nervosa, or binge-eating disorder), and can negatively affect both physical and mental health (American Psychiatric Association, 2022; Conceição and Goldschmidt, 2019).

Despite their potential clinical relevance DEBs in MS have been scarcely studied. Early case reports provide compelling evidence of the phenomenon. A case described by Touyz (1989) of a 29-year-old woman developing anorexia nervosa after a MS diagnosis at age 21 (cited in Raffman and Jayabalan, 2025). At the time of assessment, her BMI was critically low at 13.92. She exhibited emotional dysregulation, denial of her MS diagnosis, obsessive-compulsive thoughts and behaviors, and pronounced fears related to separation and death (in Raffman and Jayabalan, 2025). The onset of her eating disorder appeared closely linked to her MS diagnosis, as she intentionally restricted food intake due to fear of “feeding and strengthening her MS” and worsening her symptoms. Of note, she achieved full recovery following admission to a specialized eating disorders unit, demonstrating that multidisciplinary interventions can be effective even in the context of comorbid neurological conditions (in Raffman and Jayabalan, 2025).

Another case of disordered eating following MS diagnosis was reported by Ceschin et al. (2010). They described a 29-year-old woman who developed pica and binge eating behaviors after being diagnosed with MS at age 21. The patient exhibited impulse dysregulation, compulsive behaviors, and emotional difficulties, leading the authors to question whether the eating behaviors represented impulse dyscontrol, a compulsive symptom, or another form of eating dysregulation.

Building on these observations, studies examining DEBs in MS have reported altered eating patterns and associated psychological factors. As one of the earliest studies, Timmerman and Stuifberg (1999) examined 67 women with MS and found that altered eating patterns including restricted intake, were common and closely associated with fatigue, emotional distress, and coping difficulties, highlighting the early recognition of DEBs in this population. Recent research confirms and expands these findings, suggesting that the interplay of personality traits and self-perception difficulties contributes to vulnerability to DEBs in MS (Kiropoulos et al., 2025). Kiropoulos et al. (2025), examined 275 individuals with MS, assessing personality traits, self-esteem, body dissatisfaction, and eating disorder symptoms. They reported that 21% of participants had a lifetime diagnosis of an eating disorder, including 12.4% with anorexia nervosa, 9.5% with bulimia nervosa, and 1.5% with binge-eating disorder, while an additional 27.6% were considered at risk of developing an eating disorder. Statistically significant correlations were observed between neuroticism, low self-esteem, body dissatisfaction, and eating disorder symptoms, suggesting a complex interplay among these psychological factors.

To date, no large-scale studies have specifically investigated DEBs in adolescents with MS, representing a critical gap in the literature and an important target for future research.

## 4 Discussion

Research on multiple sclerosis has traditionally emphasized cognitive functioning, affective symptoms, and quality of life. In contrast, disturbances in bodily experience are increasingly recognized as an underexplored yet clinically significant dimension. However, research on bodily experience remains limited and largely focused on adults, with few studies on pediatric populations. (Breheny et al., 2026; Kiropoulos et al., 2025; McCormack et al., 2025; Pfaffenberger et al., 2011; Piliavska et al., 2023). Among the most frequently investigated manifestations are FNS and somatization, which often co-occur with anxiety and depression across age groups, potentially intensifying symptom perception and sustaining psychological distress (Carbonell et al., 2014; Charvet et al., 2016; Cuoco et al., 2025; Merwick and Sweeney, 2008; Piliavska et al., 2023; Till et al., 2012). These symptoms may result from the interaction of cognitive, affective, and neurobiological processes. Heightened attention to bodily sensations, maladaptive interpretation of interoceptive signals, and difficulties in emotion regulation have been identified as key mechanisms that contribute to their development and persistence (Lee et al., 2024; Raimo et al., 2024; Salamone et al., 2018). These psychological mechanisms may interact with MS-related biological factors, as inflammatory activity and lesion-related changes in neural systems involved in interoception and sensorimotor integration may contribute to atypical processing of bodily signals. Such alterations may heighten vigilance toward internal sensations and foster maladaptive interpretations, thereby potentially contributing to symptom amplification (Carotenuto et al., 2020, 2023; Raimo et al., 2024; Gonzalez Campo et al., 2020). Over time, the interaction of these mechanisms may reinforce one another, thereby contributing to the persistence and severity of FNS, somatization, and related bodily disturbances in MS (Gonzalez Campo et al., 2020; Raimo et al., 2024; Salamone et al., 2018). Another important dimension of altered bodily experience concerns disturbances in body image, reflecting the cognitive and emotional evaluation of one's own body and how it aligns with internalized ideals (Cash, 2002, 2012). MS-related symptoms, including fatigue, pain, sensory and motor impairments, and treatment side effects, may generate a discrepancy between the perceived bodily state and internalized standards. This discrepancy can undermine the sense of agency and self-continuity, contributing to body dissatisfaction and feelings of shame (Price, 1990). Beyond their direct emotional impact, these disruptions in body image may interfere with psychological adjustment to chronic illness. When bodily changes are perceived as uncontrollable or inconsistent with one's identity, individuals may experience difficulties integrating the illness into their self-concept, resulting in reduced self-acceptance and increased distress (Powell et al., 2024; White, 2000; Ziaie et al., 2024). These alterations in bodily experience may also influence coping strategies and emotional responses. Heightened attention to somatic sensations and discomfort with bodily changes can promote maladaptive responses to daily challenges and illness-related stress (Cash, 2012). In this context, dysfunctional eating behaviors may emerge as strategies to regulate negative affect or regain a sense of control over the body, with body dissatisfaction and negative affect acting as central maintaining factors (Abdoli et al., 2025). Anxiety and depression may further mediate the relationship between bodily alterations and disordered eating (Marrie et al., 2015; Sander et al., 2021), contributing to a reinforcing interplay between emotional distress, body dissatisfaction, and maladaptive behaviors. In addition to these psychological mechanisms, neurobiological alterations in MS may also play a contributory role. Changes in dopaminergic and serotonergic signaling have been proposed to influence circuits involved in interoception and reward processing, potentially contributing to fatigue, anhedonia, and emotional dysregulation (Carotenuto et al., 2020, 2023).

These alterations might also contribute indirectly to maladaptive eating behaviors (Courtiol et al., 2021); however, evidence linking these mechanisms to disordered eating in MS remains scarce, with current findings being largely indirect and derived from other clinical populations (Martin et al., 2019; Gonzalez Campo et al., 2020; Salamone et al., 2018).

A broader consideration of methodological limitations across studies on FNS, BID, and DEBs further underscores these issues, as substantial methodological heterogeneity across studies, together with limited empirical support, likely contributes to the inconsistencies observed in the literature. Overall, these discrepancies reflect differences in construct definitions, measurement approaches, sample composition, and study design. FNS and somatization have been assessed using both clinical/neurological evaluations and self-report measures (Carbonell et al., 2014; Charvet et al., 2016; Jones et al., 2018; Piliavska et al., 2023; Cuoco et al., 2025), whereas BID and disordered eating have been investigated predominantly through self-report questionnaires, often without structured diagnostic interviews or clinician-rated assessments (Kindrat, 2007; Pfaffenberger et al., 2011; Stevens et al., 2019; Lo Buono et al., 2023; Kiropoulos et al., 2025) (Table 1).

Moreover, the constructs themselves have been inconsistently conceptualized. Functional symptoms have been described using heterogeneous terminology, including FNS, functional neurological disorder, somatization, and pseudo-relapses (Stone et al., 2005; Espay et al., 2018; Carbonell et al., 2014; Walzl et al., 2022; Cuoco et al., 2025), reflecting conceptual overlap across neurological and psychiatric frameworks. In contrast, body-related outcomes relevant to body image disturbance have shown heterogeneity in conceptualization and dimensional scope, ranging from BID to closely related constructs such as body esteem (Cash, 2002; White, 2000; Davison and McCabe, 2006; Prnjak et al., 2022; Breheny et al., 2026).

These differences in conceptualization and assessment, together with the predominance of cross-sectional designs, limit direct comparisons across studies. Further heterogeneity is evident at the population level. Most studies recruited adult participants from hospital-based settings, whereas pediatric and community-based samples remain underrepresented (Till et al., 2012; Charvet et al., 2016; Tarantino et al., 2024). Samples also varied with respect to disease duration, disability level, MS phenotype, and sex distribution. Psychological distress was inconsistently assessed across studies (Table 1). Although depression and anxiety were most frequently evaluated (e.g., HADS, BDI, PHQ-9, and SCL-90-R), assessment approaches ranged from brief screening tools to more comprehensive evaluations, limiting comparability across studies (Table 1). Overall, psychological distress appears to represent a transdiagnostic dimension influencing multiple aspects of bodily experience rather than an isolated construct (Bruce and Arnett, 2008; Jones et al., 2018; Sarisoy et al., 2013; Till et al., 2012; Piliavska et al., 2023; Kiropoulos et al., 2025; Lo Buono et al., 2023; Timmerman and Stuifberg, 1999). These methodological differences likely account for inconsistent findings regarding the contribution of disease-related factors to BID. While some studies suggest stronger associations with psychosocial variables such as depression, stigma, and self-esteem, others report links with disability severity and other clinical characteristics.

Evidence is particularly scarce in POMS, which represents a small proportion of MS cases, making the recruitment of adequately powered cohorts challenging and often requiring multicenter collaboration (Jeong et al., 2019). In addition, research has traditionally focused on neurological and cognitive outcomes, with limited attention to psychosocial constructs such as body image and disordered eating (Tarantino et al., 2024). Methodological challenges are further compounded by the need to distinguish disease-related changes from normative adolescent development, as well as ethical and practical constraints in studies involving minors. The absence of routine screening for body image and eating disturbances in clinical practice may further contribute to the limited evidence base.

Adolescence represents a particularly sensitive developmental period in which bodily appearance, function, and autonomy are central to identity formation, social belonging, and self-esteem (Davison and McCabe, 2006; Meland et al., 2021). Disease onset coincides with ongoing development of body representation, identity, and eating behaviors. Consequently, disease-related physical changes, fatigue, functional limitations, and treatment effects may have a disproportionate impact on psychological adjustment. Evidence from other chronic conditions indicates that disease- or treatment-related bodily changes are associated with increased body dissatisfaction, internalizing symptoms, and psychosocial difficulties (Claytor et al., 2020; Pinquart, 2013). In adolescents with MS, perceived deviation from internalized body ideals may further amplify body image concerns and promote maladaptive coping strategies, potentially extending to BEDs. Sociocultural influences, including peer comparison and social media exposure, may further exacerbate these risks (Bonfanti et al., 2025; Jiotsa et al., 2021). Early identification of body image concerns and maladaptive eating behaviors may therefore facilitate timely multidisciplinary interventions and improve long-term outcomes.

Overall, the available evidence suggests that bodily experience may represent an important interface between neurological, psychological, and behavioral processes in MS, although findings should be interpreted cautiously due to the limited and heterogeneous nature of the literature. Evidence on BID and DEBs is scarce and mainly based on small, cross-sectional observational studies, limiting the strength and generalizability of conclusions.

Reported associations should therefore be considered preliminary, and potential publication bias, including the overrepresentation of small or positive findings, should be acknowledged. Rather than indicating definitive relationships, the current literature points to potential links between neurological changes, psychological distress, and maladaptive behavioral responses that warrant further investigation.

Within this context, this review provides an integrated overview of bodily experience in MS by synthesizing evidence on FNS, somatization, BID, and disordered eating behaviors, highlighting a biopsychosocial framework and key gaps in the literature.

## 5 Clinical implications

Although the available evidence remains preliminary, the findings of this review may have important implications for clinical practice. In addition to routine screening for anxiety, depression, and fatigue, assessing FNS, somatic symptom burden, BID, and DEBs may facilitate early identification of frequently underrecognized conditions and support timely intervention. Interventions targeting body awareness, emotion regulation, and body-related cognitions may represent promising approaches in MS. Cognitive behavioral therapy (CBT) can reduce maladaptive interpretations of bodily sensations and improve emotional regulation, and, when adapted for eating disorders, may also address dysfunctional beliefs related to body image and eating behaviors (Linardon et al., 2017; Linardon, 2018; Treasure et al., 2020).

Mindfulness-based interventions may complement these approaches by enhancing interoceptive awareness and reducing avoidance and somatic vigilance (Farb et al., 2013; Khoury et al., 2025), while body-oriented therapies-including movement-based approaches and interventions targeting sensorimotor integration-may strengthen bodily agency and recalibrate disrupted body-self processes (Bravo et al., 2024: Tarsha et al., 2020). Although these interventions have shown efficacy in populations with eating disorders or body-related difficulties, evidence in MS remains limited, and adaptations are needed to account for fatigue, motor limitations, and altered bodily perception. Additional approaches, such as interpersonal or dialectical behavior therapy, may further support emotional regulation and social functioning in patients with DEBs or body-related distress.

## 6 Limitations

Several limitations in the existing literature must be acknowledged: (1) The available literature is highly heterogeneous with respect to study design, participant characteristics, assessment methods, and outcome measures, limiting direct comparisons across studies and the possibility of drawing consistent conclusions; (2) Research examining the association between MS and DEBs is extremely scarce, and in POMS it is virtually nonexistent, limiting our understanding of prevalence, risk factors, and developmental vulnerability specific to children and adolescents. Studies on BID in pediatric-onset MS are also very limited, preventing reliable estimates of prevalence and associated risk factors (Charvet et al., 2016; Till et al., 2012); (3) Most studies on FNS and somatization have not combined clinical, neuroimaging, and interoceptive measures, providing only a partial view of mechanisms linking neurological changes to psychological disturbances (Cuoco et al., 2025; Piliavska et al., 2023; Walzl et al., 2022); (4) Research on bodily experience in MS often relies on small samples or single case reports, limiting generalizability and conclusions about prevalence or symptom profiles (Carbonell et al., 2014; Merwick and Sweeney, 2008; Pfaffenberger et al., 2011; Samonds and Cammermeyer, 1989; Till et al., 2012); (5) Studies on BID and DEBs mostly use adult cohorts and cross-sectional designs, constraining understanding of developmental trajectories and long-term adaptation (Lo Buono et al., 2023; Kindrat, 2007); (6) Sociocultural expectations, perceived stigma, and gender-specific pressures remain largely unexamined despite evidence of their role in BID and DEBs (Sengül et al., 2019; Stevens et al., 2019); (7) Many FNS studies are hospital- or clinic-based, possibly overrepresenting severe cases and not reflecting prevalence in the broader MS population (Cuoco et al., 2025; Merwick and Sweeney, 2008); (8) In addition, this review was conducted as a narrative synthesis and did not include a formal methodological quality assessment or risk-of-bias evaluation of the included studies. Consequently, the findings should be interpreted as a qualitative overview of the available evidence rather than as a graded assessment of evidence certainty. The absence of a formal quality appraisal limits the ability to evaluate the overall strength of the evidence and to draw firm conclusions; (9) The available evidence may also be affected by publication bias, as studies reporting significant or clinically notable findings are more likely to be published than null or negative results.

## 7 Future research directions

Future research should adopt longitudinal and multimodal designs integrating neuroimaging, interoceptive measures, and comprehensive psychological assessments, with a specific focus on BID and DEBs, to clarify how neurological changes contribute to disturbances in bodily experience. Greater attention to BID and BEDs across developmental stages, particularly during adolescence, is needed, given the heightened vulnerability to body-related concerns during this period. Research in pediatric-onset MS is especially important to track the evolution of these disturbances from childhood into adulthood and to inform developmentally tailored interventions; however, multicenter and collaborative studies will be crucial to overcome sample size limitations in this population. A more comprehensive understanding of bodily experience in MS may ultimately support earlier identification of psychological vulnerability and the development of more integrated, patient-centered care models.

## References

[B1] Abdoli M. Schiechtl E. Rosato M. S. Mangweth-Matzek B. Cotrufo P. Hüfner K. (2025). Body image, self-esteem, emotion regulation, and eating disorders in adults: a systematic review. Neuropsychiatr. 39, 118–132. doi: 10.1007/s40211-025-00544-4 40830328 PMC12397123

[B2] American Psychiatric Association (2022). Diagnostic and Statistical Manual of Mental Disorders: DSM-5-TR (Text Revision), 5th Edn. Arlington, VA: American Psychiatric Association. doi: 10.1176/appi.books.9780890425787

[B3] Barbagallo F. Tiranini L. Placentino C. Mariacci G. Piccinino M. Cucinella L. . (2024). Body image and other mood vulnerabilities in adolescents with polycystic ovary syndrome and metabolic alterations. Children (Basel) 11:521. doi: 10.3390/children11050521 38790516 PMC11119722

[B4] Bonfanti R. C. Melchiori F. Teti A. Albano G. Raffard S. Rodgers R. . (2025). The association between social comparison in social media, body image concerns and eating disorder symptoms: a systematic review and meta-analysis. Body Image 52:101841. doi: 10.1016/j.bodyim.2024.101841 39721448

[B5] Bravo C. Hernández-García D. Trinidad-Fernández M. Badia G. Solé S. Serrano J. (2024). Movement awareness therapies in eating disorders: a systematic review and meta-analysis. Nurs. Health Sci. 26:e13181. doi: 10.1111/nhs.13181 39438086 PMC11586511

[B6] Breheny E. O'Keeffe F. Cooney S. (2026). Body image, sexual dysfunction and psychological distress in people with multiple sclerosis. Disabil. Rehabil. 48, 152–164. doi: 10.1080/09638288.2025.2527959 40637255

[B7] Brola W. Steinborn B. (2020). Pediatric multiple sclerosis-current status of epidemiology, diagnosis and treatment. Neurol. Neurochir. Pol. 54, 508–517. doi: 10.5603/PJNNS.a2020.0069 32940341

[B8] Brown R. F. Valpiani E. M. Tennant C. C. Dunn S. M. Sharrock M. Hodgkinson S. . (2008). Longitudinal assessment of anxiety, depression, and fatigue in people with multiple sclerosis. J. Psychol. Psychother. 82, 41–56. doi: 10.1348/147608308X345614 18727845

[B9] Bruce A. S. Arnett P. A. (2008). Longitudinal study of the symptom Checklist-90-Revised in multiple sclerosis patients. Clin. Neuropsychol. 22, 46–59. doi: 10.1080/13854040601064518 18338441

[B10] Carbonell C. F. Benson L. Rintell D. Prince J. Chitnis T. (2014). Functional relapses in pediatric multiple sclerosis. J. Child Neurol. 29, 943–946. doi: 10.1177/0883073813501873 24065582

[B11] Carotenuto A. Valsasina P. Preziosa P. Mistri D. Filippi M. Rocca M. A. (2023). Monoaminergic network abnormalities: a marker for multiple sclerosis-related fatigue and depression. J. Neurol. Neurosurg. Psychiatr. 94, 94–101. doi: 10.1136/jnnp-2022-330109 36229193

[B12] Carotenuto A. Wilson H. Giordano B. Caminiti S. P. Chappell Z. Williams S. C. R. . (2020). Impaired connectivity within neuromodulatory networks in multiple sclerosis and clinical implications. J. Neurol. 267, 2042–2053. doi: 10.1007/s00415-020-09806-3 32219555 PMC7320961

[B13] Cash T. F. (2002). Cognitive-behavioral perspectives on body image. in Body Image: A Handbook of Theory, Research, and Clinical Practice, eds. T. F. Cash, and T. Pruzinsky (New York, NY: Guilford Press), 38–46.

[B14] Cash T. F. (2012). “Cognitive-behavioral perspectives on body image,” in Encyclopedia of Body Image and Human Appearance, ed. T. F. Cash (Amsterdam: Academic Press), 276–282. doi: 10.1016/B978-0-12-384925-0.00054-7

[B15] Cerutti R. Spensieri V. Valastro C. Presaghi F. (2017). A comprehensive approach to understand somatic symptoms and their impact on emotional and psychosocial functioning in children. Front. Psychol. 8, 141. doi: 10.1371/journal.pone.0171867 28178333 PMC5298337

[B16] Ceschin L. Giannunzio V. Favaro A. Santonastaso P. (2010). Pica in an eating disordered woman with multiple sclerosis: impulse dys-control, compulsive symptom or self-medication attempt? Eat. Weight Disord. 15, e116–e118. doi: 10.1007/BF03325290 20571315

[B17] Charvet L. Cersosimo B. Schwarz C. Belman A. Krupp L. B. (2016). Behavioral symptoms in pediatric multiple sclerosis: relation to fatigue and cognitive impairment. J. Child Neurol. 31, 1062–1067. doi: 10.1177/0883073816636227 26961266 PMC4925200

[B18] Claytor J. D. Kochar B. Kappelman M. D. Long M. D. (2020). Body image dissatisfaction among pediatric patients with inflammatory bowel disease. J. Pediatr. 223, 68-72.e1. doi: 10.1016/j.jpeds.2020.04.045 32711754

[B19] Conceição E. M. Goldschmidt A. (2019). Disordered eating after bariatric surgery: clinical aspects, impact on outcomes, and intervention strategies. Curr. Opin. Psychiatry. 32, 504–509. doi: 10.1097/YCO.0000000000000549 PMC6768715 31343419

[B20] Courtiol E. Menezes E. C. Teixeira C. M. (2021). Serotonergic regulation of the dopaminergic system: implications for reward-related functions. Neurosci. Biobehav. Rev. 128, 282–293. doi: 10.1016/j.neubiorev.2021.06.022 34139249 PMC8335358

[B21] Cuoco S. Scannapieco S. Barra F. Giordano C. Gregorio M. D. Barone P. . (2025). Functional neurological disorder in multiple sclerosis: a prospective study. Mult. Scler. Relat. Disord. 94:106264. doi: 10.1016/j.msard.2025.106264 39826278

[B22] Davison T. E. McCabe M. P. (2006). Adolescent body image and psychosocial functioning. J. Soc. Psychol. 146, 15–30. doi: 10.3200/SOCP.146.1.15-30 16480119

[B23] Espay A. J. Aybek S. Carson A. Edwards M. J. Goldstein L. H. Hallett M. . (2018). Current concepts in diagnosis and treatment of functional neurological disorders. JAMA Neurol. 75, 1132–1141. doi: 10.1001/jamaneurol.2018.1264 29868890 PMC7293766

[B24] Farb N. A. S. Segal Z. V. Anderson A. K. (2013). Mindfulness meditation training alters cortical representations of interoceptive attention. Soc. Cogn. Affect. Neurosc. 8, 15–26. doi: 10.1093/scan/nss066 22689216 PMC3541492

[B25] Fisher K. S. Cuascut F. X. Rivera V. M. Hutton G. J. (2020). Current advances in pediatric onset multiple sclerosis. Biomedicines 8:71. doi: 10.3390/biomedicines8040071 32231060 PMC7235875

[B26] Fornari V. Dancyger I. La Monaca G. Budman C. Goodman B. Kabo L. . (2001). Can steroid use be a precipitant in the development of an eating disorder? Int. J. Eat. Disord. 30, 118–122. doi: 10.1002/eat.1063 11439418

[B27] Gholizadeh S. Azizoddin D. R. Mills S. D. Zamora G. Potemra H. M. K. Hirz A. E. . (2019). Body image mediates the impact of pain on depressive symptoms in patients with systemic lupus erythematosus. Lupus 28, 1148–1153. doi: 10.1177/0961203319861675 31369342

[B28] Gil-González I. Martín-Rodríguez A. Conrad R. Pérez-San-Gregorio M. Á. (2020). Quality of life in adults with multiple sclerosis: a systematic review. BMJ Open 10:e041249. doi: 10.1136/bmjopen-2020-041249 33257490 PMC7705559

[B29] Göksel Karatepe A. Kaya T. Günaydn R. Demirhan A. Ce P. Gedizlioglu M. (2011). Quality of life in patients with multiple sclerosis: the impact of depression, fatigue, and disability. Int. J. Rehabil. Res. 34, 290–298. doi: 10.1097/MRR.0b013e32834ad479 21946317

[B30] Gonzalez Campo C. Salamone P. C. Rodríguez-Arriagada N. Richter F. Herrera E. Bruno D. . (2020). Fatigue in multiple sclerosis is associated with multimodal interoceptive abnormalities. Mult. Scler. 26, 1845–1853. doi: 10.1177/1352458519888881 31778101 PMC7253315

[B31] Jellinger K. A. (2024). Cognitive impairment in multiple sclerosis: from phenomenology to neurobiological mechanisms. J. Neural Transm. (Vienna) 131, 871–899. doi: 10.1007/s00702-024-02786-y 38761183

[B32] Jeong A. Oleske D. M. Holman J. (2019). Epidemiology of pediatric-onset multiple sclerosis: a systematic review of the literature. J. Child Neurol. 34, 705–712. doi: 10.1177/0883073819845827 31185780

[B33] Jiotsa B. Naccache B. Duval M. Rocher B. Grall-Bronnec M. (2021). Social media use and body image disorders: association between frequency of comparing one's own physical appearance to that of people being followed on social media and body dissatisfaction and drive for thinness. Int. J. Environ. Res. Public Health. 18:2880. doi: 10.3390/ijerph18062880 33799804 PMC8001450

[B34] Jones S. M. W. Salem R. Amtmann D. (2018). Somatic symptoms of depression and anxiety in people with multiple sclerosis. Int. J. MS Care 20, 145–152. doi: 10.7224/1537-2073.2017-069 29896052 PMC5991508

[B35] Khoury B. Tixier J. Dupuis G. (2025). A meta-analysis of the effects of mindfulness meditation training on self-reported interoception. Psychol. Med. 55, 345–362. 41198766

[B36] Kindrat S. (2007). The relationship between body image and depression in women diagnosed with relapsing remitting multiple sclerosis. Can. J. Neurosci. Nurs. 29, 8–13. 18441622

[B37] Kiropoulos L. Krug I. Dang P. L. (2025). Eating disorder symptoms in multiple sclerosis: relationships between neuroticism, body dissatisfaction, and self-esteem. Nutrients 17:1609. doi: 10.3390/nu17101609 40431352 PMC12113782

[B38] Kroenke K. (2003). Patients presenting with somatic complaints: epidemiology, psychiatric comorbidity and management. Int. J. Methods Psychiatr. Res. 12, 34–43. doi: 10.1002/mpr.140 12830308 PMC6878426

[B39] Kumar M. M. (2023). Eating disorders in youth with chronic health conditions: clinical strategies for early recognition and prevention. Nutrients 15:3672. doi: 10.3390/nu15173672 37686703 PMC10490114

[B40] Lee S. J. Lee M. Kim H. B. Huh H. J. (2024). The relationship between interoceptive awareness, emotion regulation and clinical symptoms severity of depression, anxiety and somatization. Psychiatry Investig. 21, 255–264. doi: 10.30773/pi.2023.0221 38569583 PMC10990629

[B41] Linardon J. (2018). Meta-analysis of the effects of cognitive-behavioral therapy on the core eating disorder maintaining mechanisms: implications for mechanisms of therapeutic change. Cogn. Behav. Ther. 47, 107–125. doi: 10.1080/16506073.2018.1427785 29378481

[B42] Linardon J. Wade T. D. de la Piedad Garcia X. Brennan L. (2017). The efficacy of cognitive-behavioral therapy for eating disorders: a systematic review and meta-analysis. J. Consult. Clin. Psychol. 85, 1080–1094. doi: 10.1037/ccp0000245 29083223

[B43] Lipowski Z. J. (1988). Somatization: the concept and its clinical application. Am. J. Psychiatry. 145, 1358–1368. doi: 10.1176/ajp.145.11.1358 3056044

[B44] Liu S. Y. Wrosch C. Morin A. J. S. Quesnel-Vallée A. Pruessner J. C. (2019). Changes in self-esteem and chronic disease across adulthood: a 16-year longitudinal analysis. Soc. Sci. Med. 242:112600. doi: 10.1016/j.socscimed.2019.112600 31639595

[B45] Lo Buono V. Bonanno L. Corallo F. Cardile D. D'Aleo G. Rifici C. . (2023). The relationship between body image, disability and mental health in patients with multiple sclerosis. J. Clin. Med. 12:3606. doi: 10.3390/jcm12103606 37240712 PMC10218780

[B46] Locatelli G. Matus A. James R. Salmoirago-Blotcher E. Ausili D. Vellone E. . (2023). What is the role of interoception in the symptom experience of people with a chronic condition? a systematic review. Neurosci. Biobehav. Rev. 148:105142. doi: 10.1016/j.neubiorev.2023.105142 36965864

[B47] MacAllister W. S. Boyd J. R. Holland N. J. Milazzo M. C. Krupp L. B. (2007). The psychosocial consequences of pediatric multiple sclerosis. Neurology 68, S66–S69. doi: 10.1212/01.wnl.0000259420.54635.63 17438240

[B48] Marck C. H. De Livera A. M. Weiland T. J. Jelinek P. L. Neate S. L. Brown C. R. . (2017). Pain in people with multiple sclerosis: associations with modifiable lifestyle factors, fatigue, depression, anxiety, and mental health quality of life. Front. Neurol. 8:461. doi: 10.3389/fneur.2017.00461 28928713 PMC5591834

[B49] Marrie R. A. Reingold S. Cohen J. Stuve O. Trojano M. Sorensen P. S. . (2015). The incidence and prevalence of psychiatric disorders in multiple sclerosis: a systematic review. Mult. Scler. 21, 305–317. doi: 10.1177/1352458514564487 25583845 PMC4429164

[B50] Martin E. Dourish C. T. Rotshtein P. Spetter M. S. Higgs S. (2019). Interoception and disordered eating: a systematic review. Neurosci. Biobehav. Rev. 107, 166–191. doi: 10.1016/j.neubiorev.2019.08.020 31454626

[B51] McCormack D. O'Keeffe D. F. Seery C. Eccles D. F. (2025). The association between body image and psychological outcomes in multiple sclerosis: a systematic review. Mult. Scler. Relat. Disord. 93:106226. doi: 10.1016/j.msard.2024.106226 39721211

[B52] McKay K. A. Ernstsson O. Manouchehrinia A. Olsson T. Hillert J. (2020). Determinants of quality of life in pediatric- and adult-onset multiple sclerosis. Neurology 94:e932–e941. doi: 10.1212/WNL.0000000000008667 31732567 PMC7238943

[B53] Meland E. Breidablik H. J. Thuen F. Samdal G. B. (2021). How body concerns, body mass, self-rated health and self-esteem are mutually impacted in early adolescence: a longitudinal cohort study. BMC Public Health 21:496. doi: 10.1186/s12889-021-10553-x 33711967 PMC7953559

[B54] Merwick A. Sweeney B. J. (2008). Functional symptoms in clinically definite MS—pseudo-relapse syndrome. Int. MS J. 15, 47–51. 18782499

[B55] Mey G. M. Mahajan K. R. DeSilva T. M. (2023). Neurodegeneration in multiple sclerosis. WIREs Mech Dis. 15:e1583. doi: 10.1002/wsbm.1583 35948371 PMC9839517

[B56] Mohammed E. M. A. (2024). Understanding multiple sclerosis pathophysiology and current disease-modifying therapies: a review of unaddressed aspects. Front. Biosci. (Landmark Ed) 29:386. doi: 10.31083/j.fbl2911386 39614433

[B57] Novak A. M. (2025). Growing up with MS: the adolescent experience of pediatric-onset multiple sclerosis. Adolescents 5:66. doi: 10.3390/adolescents5040066

[B58] Peres D. S. Rodrigues P. Viero F. T. Frare J. M. Kudsi S. Q. Meira G. M. . (2022). Prevalence of depression and anxiety in the different clinical forms of multiple sclerosis and associations with disability: a systematic review and meta-analysis. Brain Behav. Immun. Health 24:100484. doi: 10.1016/j.bbih.2022.100484 35856061 PMC9287158

[B59] Pfaffenberger N. Gutweniger S. Kopp M. Seeber B. Stürz K. Berger T. . (2011). Impaired body image in patients with multiple sclerosis. Acta Neurol. Scand. 124, 165–170. doi: 10.1111/j.1600-0404.2010.01460.x 21198446

[B60] Piliavska K. Dantlgraber M. Dettmers C. Jöbges M. Liepert J. Schmidt R. (2023). Functional neurological symptoms are a frequent and relevant comorbidity in patients with multiple sclerosis. Front. Neurol. 14:1077838. doi: 10.3389/fneur.2023.1077838 37114221 PMC10126263

[B61] Pinquart M. (2013). Body image of children and adolescents with chronic illness: a meta-analytic comparison with healthy peers. Body Image 10, 141–148. doi: 10.1016/j.bodyim.2012.10.008 23219705

[B62] Powell B. Mills R. Tennant A. Young C. A. Langdon D. (2024). Stigma and health outcomes in multiple sclerosis: a systematic review. BMC Neurol. 24:346. doi: 10.1186/s12883-024-03853-3 39271990 PMC11401267

[B63] Price B. (1990). A model for body-image care. J. Adv. Nurs. 15, 585–593. doi: 10.1111/j.1365-2648.1990.tb01858.x 2358576

[B64] Prnjak K. Jukic I. Mitchison D. Griffiths S. Hay P. (2022). Body image as a multidimensional concept: a systematic review of body image facets in eating disorders and muscle dysmorphia. Body Image 42, 347–360. doi: 10.1016/j.bodyim.2022.07.006 35926364

[B65] Raffman E. Jayabalan P. (2025). Disordered eating behaviors in individuals with physical disabilities: a narrative review. Am. J. Lifestyle Med. [Online]. doi: 10.1177/15598276251363516 40740659 PMC12303930

[B66] Raimo S. Ferrazzano G. Di Vita A. Gaita M. Satriano F. Veneziano M. . (2024). The multidimensional assessment of body representation and interoception in multiple sclerosis. Mult. Scler. Relat. Disord. 87:105692. doi: 10.1016/j.msard.2024.105692 38810419

[B67] Ramo-Tello C. Blanco Y. Brieva L. Casanova B. Martínez-Cáceres E. Ontaneda D. . (2021). Recommendations for the diagnosis and treatment of multiple sclerosis relapses. J. Pers. Med. 12:6. doi: 10.3390/jpm12010006 35055321 PMC8780774

[B68] Salamone P. C. Esteves S. Sinay V. J. García-Cordero I. Abrevaya S. Couto B. . (2018). Altered neural signatures of interoception in multiple sclerosis. Hum. Brain Mapp. 39, 4743–4754. doi: 10.1002/hbm.24319 30076770 PMC6866398

[B69] Samonds R. J. Cammermeyer M. (1989). Perceptions of body image in subjects with multiple sclerosis: a pilot study. J. Neurosci. Nurs. 21, 190–194. doi: 10.1097/01376517-198906000-00010 2525161

[B70] Sander J. Moessner M. Bauer S. (2021). Depression, anxiety and eating disorder-related impairment: moderators in female adolescents and young adults. Int. J. Environ. Res. Public Health. 18, 2779. doi: 10.3390/ijerph18052779 33803367 PMC7967486

[B71] Sarisoy G. Terzi M. Gümüş K. Pazvantoglu O. (2013). Psychiatric symptoms in patients with multiple sclerosis. Gen. Hosp. Psychiatry. 35, 134–140. doi: 10.1016/j.genhosppsych.2012.10.011 23260339

[B72] Sengül H. S. Sengul Y. Tak A. Z. A. Kocak M. Tunc A. (2019). Evaluation of body image perception in multiple sclerosis patients without neurological deficit. Ideggyogy. Sz. 72, 49–54. doi: 10.18071/isz.72.0049 30785246

[B73] Senkowski D. Heinz A. (2016). Chronic pain and distorted body image: implications for multisensory feedback interventions. Neurosci. Biobehav. Rev. 69, 252–259. doi: 10.1016/j.neubiorev.2016.08.009 27524638

[B74] Stevens S. D. Thompson N. R. Sullivan A. B. (2019). Prevalence and correlates of body image dissatisfaction in patients with multiple sclerosis. Int. J. MS Care. 21, 207–213. doi: 10.7224/1537-2073.2018-066 31680782 PMC6819016

[B75] Stone J. Carson A. Sharpe M. (2005). Functional symptoms and signs in neurology: assessment and diagnosis. J. Neurol. Neurosurg. Psychiatr. 76, 28–36. doi: 10.1136/jnnp.2004.061655 15718217 PMC1765681

[B76] Tarantino S. Proietti Checchi M. Papetti L. Monte G. Ferilli M. A. N. Valeriani M. (2024). Neuropsychological performances, quality of life, and psychological issues in pediatric onset multiple sclerosis: a narrative review. Neurol. Sci. 45, 1913–1930. doi: 10.1007/s10072-023-07281-y 38157101 PMC11021227

[B77] Tarsha M. S. Park S. Tortora S. (2020). Body-centered interventions for psychopathological conditions: a review. Front. Psychol. 10:2907. doi: 10.3389/fpsyg.2019.02907 32038351 PMC6993757

[B78] Till C. Udler E. Ghassemi R. Narayanan S. Arnold D. L. Banwell B. L. (2012). Factors associated with emotional and behavioral outcomes in adolescents with multiple sclerosis. Mult. Scler. 18, 1170–1180. doi: 10.1177/1352458511433918 22291032

[B79] Timmerman G. M. Stuifberg A. (1999). Eating patterns in women with multiple sclerosis. J. Neurosci. Nurs. 31, 152–158. doi: 10.1097/01376517-199906000-00004 10846646

[B80] Treasure J. Duarte T. A. Schmidt U. (2020). Eating disorders. Lancet 395, 899–911. doi: 10.1016/S0140-6736(20)30059-3 32171414

[B81] Walzl D. Solomon A. J. Stone J. (2022). Functional neurological disorder and multiple sclerosis: a systematic review of misdiagnosis and clinical overlap. J. Neurol. 269, 654–663. doi: 10.1007/s00415-021-10436-6 33611631 PMC8782816

[B82] White C. A. (2000). Body image dimensions and cancer: a heuristic cognitive behavioural model. Psychooncology 9, 183–192. doi: 10.1002/1099-1611(200005/06)9:3&lt;183::AID-PON446&gt;3.0.CO;2-L 10871714

[B83] Wolters C. Gerlach A. L. Pohl A. (2022). Interoceptive accuracy and bias in somatic symptom disorder, illness anxiety disorder, and functional syndromes: a systematic review and meta-analysis. PLoS ONE. 17:e0271717. doi: 10.1371/journal.pone.0271717 35980959 PMC9387777

[B84] Zhang X. Song Y. Wei Z. Chen X. Zhuang X. Yi L. (2023). The prevalence and risk factors of anxiety in multiple sclerosis: a systematic review and meta-analysis. Front. Neurosci. 17:1120541. doi: 10.3389/fnins.2023.1120541 37139531 PMC10149809

[B85] Ziaie L. Mazaheri M. A. Zabihzadeh A. Etemadifar M. Shokri O. Contrada R. J. (2024). Multiple sclerosis and self-alienation: a study based on self and others representations. BMC Psychol. 12:760. doi: 10.1186/s40359-024-02264-w 39696622 PMC11656560

